# Expanding Pharmacists’ Role in the Management of Non-Alcoholic Fatty Liver Disease

**DOI:** 10.3390/pharmacy11050151

**Published:** 2023-09-21

**Authors:** Majid Mufaqam Syed-Abdul

**Affiliations:** Toronto General Hospital Research Institute, University Health Network, University of Toronto, Toronto, ON M5G 1L7, Canada; majidmufaqam.syedabdul@uhn.ca

**Keywords:** NAFLD, pharmacist, roles, hepatology, community pharmacy

## Abstract

Non-alcoholic fatty liver disease (NAFLD) stands as an increasingly pressing global health challenge, underscoring the need for timely identification to facilitate effective treatment and prevent the progression of chronic liver disorders. Given the projected scarcity of specialized healthcare professionals, particularly hepatologists and gastroenterologists, the role of pharmacists emerges as pivotal in NAFLD management. This article sheds light on the potential of pharmacists within community pharmacy settings, not as diagnostic entities, but as facilitators in recognizing and screening individuals at elevated NAFLD risk using validated non-invasive tools like portable devices and calculators. By prioritizing patient education, referrals, and continuous monitoring, pharmacists can refine NAFLD management, ultimately advancing patient outcomes. Enhancing pharmacists’ impact in early NAFLD detection and management can be facilitated through collaborations with healthcare institutions and the incorporation of patient self-assessment tools. This collaborative approach holds promise for further promoting improved liver health within the community.

## 1. Background

Non-alcoholic fatty liver disease (NAFLD) is characterized by an increased lipid content in the liver, i.e., ≥5.5% of liver volume estimated via histology or imaging. A more severe form of NAFLD involves the presence of inflammation as well as cell death [1,2,3,4]. Currently, 25–38% of the population exhibits characteristics of NAFLD [5,6,7], and the rates are increasing, with an estimated 4613 cases per 100,000 person-years. The incidence rate is higher in males compared to females and in overweight/obese individuals compared to those with normal weight [8,9]. In parallel with the rise in obesity, the prevalence of NAFLD is significantly contributing to the growing burden of chronic liver disease worldwide [5,6,9,10,11,12,13,14]. An advanced form of the disease, nonalcoholic steatohepatitis (NASH), has increased 2.0–2.5-fold recently [6,15] and was significantly associated with liver-related morbidity [16,17]. Furthermore, NASH was predicted to become the second most prevalent cause of liver transplantation [18].

## 2. NAFLD Stages and Clinical Manifestations

NAFLD is categorized into four stages based on the presence of pathological characteristics [1,2,3,4,19,20]. Although the progression of NAFLD does not necessarily occur in the order presented here, the initial stage of NAFLD, called hepatic steatosis, is characterized by an increased accumulation of intrahepatic triacylglycerols (IHTG) in the liver [4,17,21,22,23,24,25]. This phenomenon predominantly stems from aberrant hepatic lipid metabolism, regardless of the presence or absence of obesity [6,10,26,27,28,29,30,31,32,33].

Studies conducted in humans have shown that 20% of cases of NAFLD develop increased inflammation and cellular stress, resulting in an advanced form of the disease known as NASH, which is the second stage of the disease [21,23,24,34,35,36,37,38,39]. NASH has been documented as an increasingly prevalent factor in the development of hepatocellular carcinoma (HCC) among patients who are undergoing liver transplantation [28].

Approximately 13–25% of NASH patients can progress to the third stage, called fibrosis, which involves scarring of the liver tissue [37]. This scarring can lead to irreversible damage and, subsequently, to cirrhosis of the liver in 2–12% of patients, representing the fourth stage of NAFLD, and eventually leading to HCC [1,4,17,19,23,33,37,40].

## 3. Significance of Diagnosing NAFLD in the Early Stages

In clinical practice, the early detection of NAFLD holds paramount importance to facilitate the most effective treatment [41]. While approved treatments for NAFLD are currently lacking, a range of investigational drugs targeting different stages of the disease are in development. Nonetheless, as NAFLD advances to stage 4 (cirrhosis), liver transplantation becomes the primary treatment option, despite the use of multiple medications, such as antibiotics, antivirals, beta-blockers, and angiotensin-converting enzyme (ACE) inhibitors, for cirrhosis-associated complications [42]. Therefore, the timely diagnosis of NAFLD at its earliest stage assumes critical significance.

## 4. Opportunities for Pharmacists in the Management of NAFLD

With the current increasing incidence of NAFLD and the reported shortage of general physicians [43,44,45,46,47,48] as well as the predicted scarcity of gastroenterologists and hepatologists in the near future [49], pharmacists find themselves with a unique opportunity to undertake a new role in the early detection and management of NAFLD within the general population. Due to their easy accessibility to the public and availability in community pharmacies 24/7, pharmacists possess a significant advantage over other healthcare professionals in detecting and managing NAFLD in outpatient or community settings. Although it requires proper education and in-depth knowledge of the disease, this article presents an approach to assist pharmacists in initiating and developing NAFLD education and management programs akin to existing diabetes programs [50]. Employing simple terms like “Liver Health Clinic” or “MyLiver Health” could be utilized to capture the attention of patients visiting pharmacies to pick up their prescriptions. The primary goal of such programs, making pharmacists critical in solving, would be to overcome the crisis of the increased NAFLD epidemic across the world.

## 5. Patient Identification

Given the increased incidence of NAFLD at approximately 25% before 2015 and approximately 38% after 2016, with an overall rate of 46.9 cases per 1000 person-years and 70.8 cases per 1000 person-years in men alone [7], pharmacists play a crucial role in identifying individuals (approximately 38% of the global population) with NAFLD. To enhance the success rates of NAFLD programs, pharmacists can begin by focusing on individuals at high risk of developing the condition. The following list comprises anthropometric, family history-related, and biochemical factors that can be used to identify patients suitable for the program. Additionally, pharmacists can initiate conversations by inquiring about the patient’s prescription medication history. For example, patients taking medications for dyslipidemia, diabetes, or being overweight may be at increased risk of NAFLD. In such cases, pharmacists can introduce the program verbally or use preprinted educational brochures and invite these patients to monitor their liver health. Once patients agree to participate, pharmacists can employ the following approach to detect the presence of NAFLD or any other form of liver disease and take appropriate steps accordingly.

## 6. Screening for NAFLD in the Outpatient Pharmacy

Evaluating the health status of potential patients with NAFLD can be challenging for pharmacists, let alone developing a short- and long-term plan for improvement, particularly in outpatient or community pharmacies where pharmacists do not have access to their laboratory results. The simplest approach would be to create a questionnaire that encompasses the major factors previously shown to predict NAFLD. While researchers and physicians may have access to invasive measurements, pharmacists must carefully balance their choices by selecting non-invasive yet essential variables that can predict NAFLD with greater confidence. Given the practical challenges in outpatient or community pharmacies and the limitations pharmacists may face, presented below are some tools that can be utilized by them.

Once patients are suspected of having NAFLD, a report can be generated and sent to their primary care physicians for a detailed check-up. At this point, pharmacists should also take the opportunity to validate and compare their results with the more sophisticated and direct diagnostic methods used in physicians’ offices or hospitals.

### 6.1. Common Variables to Identify NAFLD

Numerous variables with proven predictive capabilities for NAFLD have been documented in the existing literature. Nevertheless, it is essential to acknowledge that, within a community pharmacy environment, it may not be feasible to employ all the variables. Consequently, it becomes imperative for pharmacists to discern the pharmacy’s resource limitations and identify suitable variables that can be efficiently and accurately measured.

In addition to the variables listed in Table 1, pharmacists should also aim to collect some of the non-invasive variables like anthropometrics and family history.

**Anthropometrics and physical examination:** In community settings, among the most prevalent and easily obtainable measurements are height, weight, waist, and hip measurements. Height and weight serve as the basis for calculating body mass index (BMI), while waist and hip measurements enable the computation of the waist-hip ratio. Both BMI and waist-hip ratio possess significant predictive value concerning metabolic syndrome, a commonly observed characteristic in NAFLD [6,51,52]. Pharmacists may also consider quantifying body fat percentage over BMI, since it has been shown to predict NAFLD [53]. While dual-energy X-ray absorptiometry is the gold standard, other less expensive tools have been validated to predict body fat percentage [54]. Lastly, while practically and legally not possible for pharmacists in many countries, some countries may allow pharmacists to physically examine patients. However, it is important to note that these examinations require additional resources that pharmacists should consider before offering such services at their pharmacy.

Moreover, when resources and technical expertise are available, indirect tools (e.g., bioelectrical impedance analysis, BIA [55]) can be employed by pharmacists to assess body composition, offering additional insights into an individual’s health status and potential risk factors. The adoption of these measurements and assessments can contribute to a more comprehensive evaluation of patients’ well-being and facilitate the early detection of health concerns related to NAFLD and metabolic syndrome.

**Family history:** Family history plays a crucial role in predicting susceptibility to non-alcoholic fatty liver disease (NAFLD). Inherited factors passed down through generations significantly influence an individual’s likelihood of developing NAFLD. A positive family history of metabolic disorders, obesity, type 2 diabetes, and dyslipidemia elevates the risk of NAFLD occurrence [56,57]. Accurate identification of familial predisposition enables pharmacists to proactively monitor individuals at higher risk. Therefore, understanding these hereditary factors and by incorporating a family history assessment with other pertinent risk factors and biomarkers, pharmacists can optimize personalized preventive strategies and enhance patient care for NAFLD.

**Figure 1:**
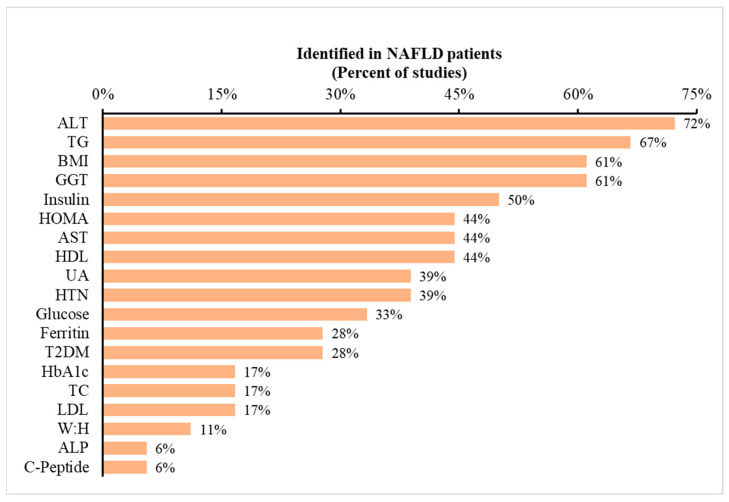
Occurrence of metabolic variables reported in prior studies of NAFLD patients. Legend: The dataset consists of 17 studies. The data depict the percentage of studies reporting specific metabolic variables listed on the *x*-axis. To illustrate, 72% of previous studies indicated ALT as a metabolic marker for predicting NAFLD. Abbreviations: ALT: alanine aminotransferase; BMI: body mass index; AST: aspartate aminotransferase; UA: uric acid; T2DM: type 2 diabetes mellitus; TC: total cholesterol; and W:H: waist-to-hip ratio.

**Blood biomarkers:** Based on the previous literature, 17 studies involving NAFLD patients were identified and evaluated for blood biomarkers [58,59,60,61,62,63,64,65,66,67,68,69,70,71,72]. A total of 19 blood biomarkers were recognized that were altered in the presence of NAFLD. These biomarkers are listed in Figure 1 in the order of their frequency. For example, alanine transaminase (ALT) was reported to be increased in NAFLD patients in 72% of the studies included in the analysis, whereas only 6% of the studies reported increases in alkaline phosphatase (ALP) and c-peptide. Although limited within the settings of a community pharmacy, these blood biomarkers offer valuable insights into potential NAFLD diagnoses. Pharmacists with access to patients’ medical records or connectivity to pathology labs can utilize this information for informed clinical decision making (i.e., potentially identifying underlying NAFLD).

### 6.2. Non-Invasive or Minimally Invasive Tools

Considering the invasiveness and technical requirements of several listed variables, pharmacists can explore non-invasive alternatives for assessing NAFLD risk within the community pharmacy setting. Utilizing portable devices or scientifically validated calculators offers a more practical approach, requiring minimal training and enabling quick assessments. While these non-invasive methods may not provide the same level of precision as laboratory-based measurements, they offer a convenient snapshot of a patient’s health status. By incorporating these tools into their practice, pharmacists can efficiently screen for NAFLD risk and enhance early detection in their patients.

Listed below are some examples of portable devices and calculators that can be utilized in community pharmacy settings:

**Portable devices:** The examples of portable devices mentioned here aim to broaden the pharmacist’s awareness of available resources. It is essential to acknowledge that there might be other validated options specifically designed for liver measurements.

*Non-Invasive Liver Fat and Fibrosis Assessment:* Devices such as FibroScan (Echosens, Paris, France) or elastography can assess and predict liver fat scores and fibrosis without the need for a liver biopsy [73]. FibroScan allows for the rapid and painless evaluation of liver health, offering valuable insights into disease progression. By using transient elastography, FibroScan measures liver stiffness, indicating fibrosis severity, and controlled attenuation parameter (CAP), which estimates liver fat content. FibroScan’s benefits include its non-invasive nature, quick assessments, and real-time results. However, it is essential to consider its limitations, such as operator dependency and potential inaccuracies in certain patient populations. Despite these considerations, FibroScan remains a valuable resource for non-invasive liver assessments and aids in enhancing patient care for liver-related conditions.

*Portable Ultrasound:* Similar to regular ultrasound, a portable device like the Vscan Extend Dual Probe by GE Healthcare (Chicago, IL, USA) can provide a non-invasive assessment of hepatic steatosis and liver health. This portable ultrasound device offers advantages such as portability, real-time imaging, non-invasiveness, quick assessments, and a user-friendly interface, requiring minimal training. However, pharmacists should consider the higher upfront costs, limited diagnostic accuracy compared to high-end machines, and operator dependency. Other factors to consider include the device’s limited depth penetration and image quality. Despite these limitations, portable ultrasound provides valuable insights into liver health, making it a potentially useful tool for community pharmacy settings.

*Portable Blood Biomarker Testing Kits:* Several blood biomarker portable and quick testing kits are available in the market for pharmacists to consider. The A1CNow+ by PTS Diagnostics (Indianapolis, IN, USA) measures hemoglobin A1c (HbA1c). The CardioChek Plus by Polymer Technology Systems (Indianapolis, IN, USA) allows for the rapid assessment of multiple parameters, including total cholesterol, HDL cholesterol, triglycerides, and glucose. The Nova StatStrip Glucose/Ketone by Nova Biomedical (Waltham, MA, USA) enables simultaneous measurements of blood glucose and ketones. While these testing kits can offer rapid results at the point of care, facilitating immediate insights into biomarker levels, their analytical accuracy may be slightly limited compared to sophisticated laboratory equipment, potentially leading to marginally less precise results. Despite this drawback, these kits can provide a valuable and convenient resource for pharmacists to swiftly assess biomarkers and aid in timely patient care and management.

**Calculators:** In addition to the direct measurements presented above, pharmacists may also utilize indirect predictors of NAFLD. The calculators presented below were based on studies in clinical settings, thus providing some confidence to the pharmacist. While not mandatory, pharmacists may choose to utilize one or more calculators in their practice; however, this necessitates measuring or collecting additional variables as required by each calculator. If a pharmacist can obtain all 17 variables, he or she can perform all the NAFLD-predicting calculations, encompassing age, sex, ethnicity, race, BMI, diabetes mellitus (DM) status, hypertension (HTN) status, metabolic syndrome (MetS), TG, glucose, fasting insulin, albumin, platelet count, AST, ALT, CAP, and liver stiffness measurement (LSM). Below is the list of calculators presented in Table 2 that can be used by pharmacists.

### 6.3. Feasibility of Assessment

Assessing NAFLD in community pharmacy settings presents certain challenges and opportunities. The feasibility of this assessment revolves around the availability of resources and the practicality of implementing non-invasive tools. While most community pharmacists lack access to specialized laboratory equipment, they can adopt alternative approaches to efficiently screen for NAFLD risk factors. For example, sophisticated devices like FibroScan cost tens of thousands of dollars, which most pharmacies cannot afford. Additionally, they also require expertise or training to operate the device. Therefore, only pharmacies with a larger budget can consider employing such devices. Alternatively, other devices like portable ultrasound or biomarker testing kits provide less expensive options for pharmacies to consider. Lastly, to enhance feasibility, pharmacists can employ a screening questionnaire that includes key risk factors and biomarkers associated with NAFLD. Although this approach may not encompass all variables, it allows for the identification of high-risk individuals, streamlining targeted assessments.

Non-invasive portable devices and calculators offer practical solutions for community pharmacy settings. Devices like FibroScan and portable ultrasounds provide rapid liver health evaluations, offering valuable insights into disease progression. While their diagnostic accuracy may differ from clinical and laboratory-based methods, these devices present convenient and timely assessments, complementing patient care. Additionally, portable blood biomarker testing kits can offer rapid results for glucose, lipids, and liver enzymes, providing valuable insights into potential NAFLD diagnoses. Pharmacists can collaborate with nearby healthcare facilities to access more comprehensive testing capabilities, optimizing diagnostic accuracy.

Overall, with the right approach and resource management, community pharmacists can make substantial contributions to NAFLD assessment, early detection, and improved patient care within their settings. Their proactive role in implementing non-invasive tools and targeted screening questionnaires demonstrates the potential for community pharmacies to play a crucial part in the early detection and management of NAFLD, ultimately enhancing patient outcomes and overall public health.

### 6.4. Limitations and Alternate Solutions

**Limitations:** The specific roles of pharmacists in NAFLD management might vary depending on the health system, the availability of resources, the extent of collaboration, and, in particular, the practice of pharmacy. Despite the potential for community pharmacists to play a significant role in NAFLD management, there are certain limitations to be acknowledged. One primary limitation lies in the lack of access to specialized laboratory equipment within community pharmacy settings. While non-invasive portable devices and calculators offer practical alternatives, they may not provide the same level of precision as laboratory-based assessments. Consequently, the diagnostic accuracy of NAFLD screening in community pharmacies may be marginally reduced compared to assessments conducted in specialized healthcare facilities.

Furthermore, the implementation of NAFLD assessment and management programs in community pharmacies may require additional resources and training for pharmacists. Time constraints within busy pharmacy environments may limit the extent to which pharmacists can conduct comprehensive assessments or patient education. Additionally, the availability of qualified personnel to operate portable devices and interpret results accurately may pose a challenge.

Moreover, community pharmacists may face limitations in directly diagnosing NAFLD, as a definitive diagnosis often requires specialized medical evaluation and advanced imaging techniques, which may not be available within the pharmacy setting. As a result, community pharmacists must emphasize the importance of timely referrals to primary care physicians, gastroenterologists, or hepatologists for further evaluation and definitive diagnosis.

Lastly, it is important to note that most of the suggestions provided in this review are based on the literature presented in studies conducted in a clinical setting. To the best of our knowledge, no studies have been conducted within a community pharmacy setting. Thus, it is imperative to recognize that the efficacy of these tools in accurately predicting NAFLD within community pharmacy settings might be limited.

**Alternate Solutions:** *Collaborative Partnerships*: Community pharmacists can establish collaborative partnerships with nearby healthcare facilities or laboratories to gain access to more comprehensive testing capabilities. By working together with these institutions, pharmacists can enhance the diagnostic accuracy of NAFLD assessments and ensure timely referrals for further evaluation and specialized care.

*Patient Self-Assessment Tools*: To overcome the limitations of time constraints and resource availability, community pharmacists can develop patient self-assessment tools or questionnaires that encompass key risk factors and biomarkers associated with NAFLD. Patients can complete these assessments in the pharmacy or at home, providing valuable insights that can guide pharmacists in identifying high-risk individuals and facilitating appropriate follow-up.

*Telehealth Services*: Incorporating telehealth services within community pharmacies can broaden the reach of NAFLD management. Pharmacists can collaborate with remote healthcare providers, including gastroenterologists and hepatologists, to conduct virtual assessments and consultations with patients, enabling timely diagnosis and personalized care plans.

*Program’s Evaluation*: Given the lack of studies conducted within a community pharmacy setting, it is prudent to consider the potential for leveraging the expertise of pharmacists in offering personalized consultations and guidance. While the predictive accuracy of existing tools might be uncertain in this context, community pharmacists could potentially play a role in utilizing their clinical judgment and collaborating with healthcare professionals to identify individuals at risk of NAFLD based on a holistic assessment of lifestyle factors, medical history, and relevant biomarkers. This approach could underscore the unique position of pharmacists in NAFLD identification, prevention, and management within their local communities. Additionally, it presents an opportunity to generate data for potential research studies aimed at evaluating the efficacy of such programs.

## 7. Patient Education, Referral, Lifestyle and Therapeutic Guidance, and Follow-Up

When patients exhibit abnormal NAFLD results obtained through questionnaires, blood tests, quick test kits, portable devices, or NAFLD calculators in the community pharmacy setting, pharmacists can play a pivotal role in patient education/counseling, facilitating appropriate referrals, and encouraging timely follow-up with healthcare providers. By empowering patients with knowledge and facilitating the continuum of care, pharmacists contribute significantly to the early detection and effective management of NAFLD, ultimately improving patient outcomes and overall health in the community.

### 7.1. Patient Education/Counseling

Upon receiving abnormal NAFLD results, pharmacists should engage in patient education and counseling to ensure that individuals understand the implications of their test outcomes. Pharmacists can explain the significance of NAFLD, its potential health risks, and the importance of early detection and management. Patients need to be informed about lifestyle modifications, such as adopting a balanced diet, engaging in regular physical activity, and maintaining a healthy weight, as these can significantly impact NAFLD progression [4]. Additionally, pharmacists should counsel patients on avoiding alcohol consumption and the potential adverse effects of certain medications on the liver [6,7,60]. Emphasizing the importance of regular follow-up with healthcare providers is essential for effective NAFLD management.

### 7.2. Referral

For patients with abnormal NAFLD results, pharmacists must facilitate appropriate referrals to primary care physicians, gastroenterologists, or hepatologists for further evaluation and management. Although pharmacists may not have the authority to diagnose NAFLD definitively, they can act as advocates for their patients’ health by ensuring that they receive comprehensive assessments and follow-up care from qualified healthcare professionals. Referral to specialists will enable patients to undergo more in-depth investigations, such as liver imaging or biopsy, and receive personalized treatment plans based on their specific NAFLD stage and risk factors.

### 7.3. Lifestyle and Therapeutic Guidance

Pharmacists can offer essential lifestyle (diet and physical activity) and therapeutic guidance to individuals with potential NAFLD. Although this requires significant educational learning, and because there is some interest in pharmacy professionals to educate themselves about nutrition [83], pharmacists can educate patients on basic dietary modifications like adopting a balanced diet with an emphasis on fruits, vegetables, lean proteins, and portion control, while minimizing saturated fats and refined sugars. Additionally, pharmacists can encourage these patients to engage in regular physical activity, including both aerobic exercises and strength training, to aid in weight management. Pharmacists also can stress the importance of medication adherence, regular monitoring of liver function, and collaborating with healthcare providers to manage associated conditions like diabetes and hypertension. Lastly, although some states in the US are now allowing pharmacists to prescribe certain medications, pharmacists must pay caution before prescribing any medication. It is critical to note that the main proposed new role of the pharmacist here is to identify, rather than treat, potential NAFLD patients.

### 7.4. Follow-Up

Following referral, pharmacists should encourage patients to diligently attend their appointments with healthcare providers. Patients may feel anxious or uncertain about their condition, and regular follow-up with physicians can provide reassurance and a clearer understanding of their NAFLD status. Pharmacists can offer continuous support and monitoring during the follow-up process, addressing any concerns or questions patients may have. Additionally, pharmacists can collaborate with healthcare providers to stay informed about their patients’ progress and treatment plans, ensuring seamless care coordination.

## 8. Resources

### 8.1. Resources for Pharmacists

Pharmacists can benefit from various resources to effectively manage NAFLD. They can access comprehensive NAFLD educational materials covering its pathophysiology, risk factors, diagnostics, and management strategies through reputable organizations and academic institutions. Engaging in continuing education programs focused on NAFLD will enhance their knowledge and competence in the latest developments and research. Clear referral guidelines and resources facilitating patient referrals to specialized healthcare providers will aid in seamless care coordination. Establishing strong communication channels with primary care physicians, gastroenterologists, hepatologists, and other healthcare providers will foster collaboration in patient management. Additionally, educational webinars, conferences, and workshops specific to NAFLD can keep pharmacists updated on emerging trends and best practices. Access to professional networks and online forums dedicated to NAFLD will enable them to seek guidance and share experiences with their peers.

### 8.2. Resources for Patients

Pharmacists should plan to design and provide several resources to potential patients with NAFLD to manage their condition effectively. Patient-friendly brochures or pamphlets explaining NAFLD, its risks, and lifestyle changes will help them better understand the condition and the importance of proactive management. Access to dietary guidelines tailored to NAFLD patients will support them in making healthier food choices and managing their weight. Resources encouraging regular physical activity and offering suitable exercise routines will aid in adopting active lifestyles, contributing to better overall health. Support groups or online forums where patients can connect with others facing similar challenges in managing NAFLD will provide emotional support, motivation, and valuable insights into coping strategies. Additionally, reminders and alerts for scheduled appointments with healthcare providers will ensure timely follow-up and continuity of care, optimizing patient outcomes and promoting proactive management of NAFLD.

## 9. Potential Impact

Community pharmacists can make a substantial contribution to addressing the NAFLD epidemic by providing education and raising awareness about NAFLD risk factors and prevention, reviewing medication profiles to identify potential contributors to liver health issues, offering personalized lifestyle counseling for dietary changes and exercise routines, implementing screening tools to identify at-risk individuals and referring them to appropriate healthcare professionals, collaborating with physicians to ensure comprehensive NAFLD management, promoting vaccination against diseases impacting liver health, establishing follow-up appointments to monitor progress and provide ongoing support, and collecting patient feedback to gauge the effectiveness of their interventions. Key metrics for evaluating their impact include the number of educated patients, referrals made, medication reviews conducted, counseling sessions held, vaccination rates, follow-up compliance, and patient feedback, all of which collectively showcase their role in improving liver health outcomes.

## 10. Conclusions

In conclusion, this article emphasizes the vital role of pharmacists in managing NAFLD within a community pharmacy setting. The escalating incidence of NAFLD underscores the importance of early detection to facilitate effective treatment and reduce the burden of chronic liver disease. Given the predicted scarcity of specialized healthcare professionals in liver diseases, including gastroenterologists and hepatologists, empowering pharmacists with the necessary knowledge and tools to assume a new role in NAFLD management becomes even more critical and presents a promising opportunity. The article underscores the significance of patient identification and screening, employing non-invasive tools like portable devices and scientifically validated NAFLD calculators. By utilizing variables such as anthropometrics, family history, and blood biomarkers, pharmacists can effectively identify individuals at high risk of developing NAFLD. By collaborating with other healthcare professionals and adopting a proactive approach, pharmacists can make significant contributions to the early detection and effective management of NAFLD, ultimately improving patient outcomes and advancing liver health within the community.

## Figures and Tables

**Table 1:** Known factors and rationale for using them for NAFLD diagnosis.

Variables	Rationale
Prior diagnosis	Individuals previously diagnosed with NAFLD.
Ethnicity	African Americans exhibit lower susceptibility to NAFLD.
High AST, plasma TG, BMI, GGT, insulin, low HDL, high HOMA-IR score	Patients exhibiting these characteristics were more prone to having a severe form of NAFLD.
High HbA1c	Diabetics are more prone to NAFLD, thus HbA1c screening can be performed.

**Table 2:** NAFLD Calculators.

Calculator Name	Formula
Aspartate Aminotransferase to Platelet Ratio Index (APRI) [74]	APRI = (AST (U/L)/upper limit normal) × 100/platelet 10^9^/L
BARD Score [75]	BARD = 1 point (if BMI > 28) + 2 points if AST/ALT ratio > 0.8 + 1 point (if diabetic)
Dallas Steatosis Index (DSI) [76]	DSI = −9.388 + 0.316 (if ≥50 years of age and female) + 2.43 (if known diabetes) + 0.019 × (equals 0 if diabetic; if not diabetic equals the glucose concentration in mg/dL) + 0.288 (if known hypertension) + 0.495 (if Hispanic/Asian/other race/ethnicity) + Ln (triglycerides in mg/dL) + 0.408 (if alanine aminotransferase [ALT] 13.5–19.49 IU/L) + 1.107 (if ALT 19.5–40 IU/L) + 1.515 (if ALT >40 IU/L) + 0.692 (if not black and BMI 25–27.49 kg/m^2^) + 1.429 (if not black and BMI 27.5–34.9 kg/m^2^) + 1.933 (if not black and BMI 35–37.49 kg/m^2^) + 2.643 (if not black and BMI >37.5 kg/m^2^) − 0.163 (if black and BMI 25–27.49 kg/m^2^) + 0.882 (if black and BMI 27.5–34.9 kg/m^2^) + 0.759 (if black and BMI 35–37.49 kg/m^2^) + 1.806 (if black and BMI >37.5 kg/m^2^)
FibroScan-AST (FAST) Score [77]	FAST = (EXP(−1.65 + 1.07 × LN(LSM) + 2.66 × 10^−8^ × (CAP)^3^ − 63.3 × (AST U/L)^−1^))/(1 + EXP(−1.65 + 1.07 × LN(LSM) + 2.66 × 10^−8^ × (CAP)^3^ − 63.3 × (AST)^−1^))
Fibrosis index based on the 4-factors (FIB-4) [78]	FIB-4 = [age (years) × AST (U/L)]/[platelet (10^9^/L) × √(ALT (U/L))]
Framingham Steatosis Index (FSI) [79]	FSI = −7.981 + 0.011 × age (years) − 0.146 × sex (female = 1, male = 0) + 0.173 × BMI (kg/m^2^) + 0.007 × triglycerides (mg/dL) + 0.593 × hypertension (yes = 1, no = 0) + 0.789 × diabetes (yes = 1, no = 0) + 1.1 × ALT/AST ratio ≥ 1.33 (yes = 1, no = 0)
Hepatic Steatosis Index (HSI) [80]	HSI = 8 × ALT/AST ratio + BMI + 2 (if DM) + 2 (if female)
NAFLD Fibrosis Score (FS) [81]	FS = −1.675 + [0.037 × age (years)] + [0.094 × BMI (kg/m^2^)] + [1.13 × hyperglycemia or diabetes (yes = 1)] + [0.99 × AST/ALT ratio] − [0.013 × platelet (×10^9^/L)] − [0.66 × albumin (g/dL)]
NAFLD Liver Fat Score (LFS) [82]	LFS = −2.89 + 1.18 × metabolic syndrome (yes = 1/no = 0) + 0.45 × type 2 diabetes (yes = 2/no = 0) + 0.15 × fasting insulin (U/L) + 0.04 × fasting AST (U/L) − 0.94 × AST/ALT

## Data Availability

The data presented in this study are available on a reasonable request from the corresponding author.
